# Complete Genome Sequence of Mycobacterium heckeshornense JCM 15655^T^, Closely Related to a Pathogenic Nontuberculous Mycobacterial Species, Mycobacterium xenopi

**DOI:** 10.1128/MRA.00020-21

**Published:** 2021-03-11

**Authors:** Mitsunori Yoshida, Hanako Fukano, Takanori Asakura, Masato Suzuki, Yoshihiko Hoshino

**Affiliations:** a Department of Mycobacteriology, Leprosy Research Center, National Institute of Infectious Diseases, Tokyo, Japan; b Antimicrobial Resistance Research Center, National Institute of Infectious Diseases, Tokyo, Japan; Georgia Institute of Technology

## Abstract

Mycobacterium heckeshornense is a slow-growing mycobacterial species for which pathogenic features are unclear. Here, we report the complete genome sequence of an *M. heckeshornense* type strain. This sequence will provide essential information for future taxonomic and comparative genome studies of these mycobacteria.

## Abstract (precis)

Mycobacterium heckeshornense is a slow-growing mycobacterial species for which pathogenic features are unclear. Here, we report the complete genome sequence of a *M. heckeshornense* type strain. This sequence will provide essential information for future taxonomic and comparative genome studies of these mycobacteria.

## ANNOUNCEMENT

Mycobacterium heckeshornense is a slow-growing nontuberculous mycobacterial (NTM) species. It was originally isolated from an immunocompetent patient with severe cavitary lung disease (1). *M. heckeshornense* is most closely related to M. xenopi (1, 2), a species that is isolated from patients in European countries and has a relatively high mortality compared with that of other NTM pulmonary diseases (3–5). In contrast, *M. heckeshornense* is rarely isolated, and little is known about its pathogenetic features. Here, we report the complete genome sequence of *M. heckeshornense* strain JCM 15655^T^, which helps us to understand characteristics of this pathogen.

*M. heckeshornense* strain JCM 15655^T^ (=CCUG 51897, =CIP 107347, =DSM 44428) was purchased from a biobank (Japan Collection of Microorganisms). The strain was inoculated on 2% Ogawa medium (Kyokuto, Tokyo, Japan) and incubated at 37°C for 4 weeks. Genomic DNA was extracted by a standard phenol-chloroform method (6, 7). Long-read data (228,122 reads) were obtained with the MinION device (Oxford Nanopore Technologies, Oxford, UK). Approximately 140 ng of the genomic DNA was used for library preparation with the SQK-RAD004 rapid barcoding sequencing kit in accordance with the manufacturer’s protocol. The library was sequenced using a SpotON Mk I (R9.4) flow cell and MinKNOW version 19.12.2. Raw sequence data were base called using guppy version 4.2.3 software. Reads shorter than 700 bp were filtered using Filtlong software (https://github.com/rrwick/Filtlong). The remaining reads (140,880 reads and a read length *N*_50_ of 12,584 bp) were *de novo* assembled into 1 contig with the “suggestCircular” flag, using Canu version 1.8 (8) with the following parameters: ContigFilter 50 10000 1.0 0.5 20 and genomeSize 5 M. The assembled genome was circularized by manually trimming the repeated sequences. Using the same genomic DNA sample, Illumina paired-end (2 × 300 bp) reads (754,736 reads) were obtained with the MiniSeq system (Illumina, San Diego, CA). The DNA library was prepared for sequencing of Illumina reads using the Nextera XT DNA library kit (Illumina) according to the manufacturer’s instructions. Low-quality reads were trimmed using sickle (https://github.com/najoshi/sickle) with the following parameters: -q 30 and -l 20. The quality of trimmed reads was checked using FastQC version 0.11.8 (http://www.bioinformatics.babraham.ac.uk/projects/fastqc/). Remaining reads (576,287 reads) were mapped to the assembly using the BWA aligner version 0.7.17 (9) for sequence and assembly error correction with Pilon version 1.2.3 (10). The resulting sequence was annotated using DFAST pipeline version 1.1.15 (11), and average nucleotide identity (ANI) was calculated by JSpeciesWS version 3.3.0 (12), with default settings.

The chromosome of *M. heckeshornense* JCM 15655^T^ is 4,957,188 bp (G+C content 65.9%). The ANI values with respect to two reported draft genomes of *M. heckeshornense* strain RLE (13) and strain CTRI-134 were 99.85% and 99.43%, respectively. Also, the ANI values with respect to complete genomes of M. xenopi strain JCM 15661^T^ (14) and *M. noviomagense* strain JCM 16367^T^ (2), which are the mycobacterial species phylogenetically closest to *M. heckeshornense*, were 89.23% and 82.88%, respectively, confirming the taxonomic position of *M. heckeshornense*. The number of predicted coding sequences, rRNA operons, and tRNAs in the genome were 4,842, 6, and 45, respectively. The complete genome sequence of *M. heckeshornense* JCM15655^T^ comprises essential data for future taxonomic and comparative genome studies.

### Data availability.

The genome sequence and annotations of *M. heckeshornense* were deposited at DDBJ/EMBL/GenBank under the accession number AP024237. Raw sequence data for strain JCM 15655^T^ were deposited under DRA accession numbers DRX250621 (MinION) and DRX250620 (MiniSeq).
